# Higher drinking frequency corresponds to lower synaptic density in people with alcohol use disorder

**DOI:** 10.1172/JCI199989

**Published:** 2026-01-13

**Authors:** Yasmin Zakiniaeiz, Nakul R. Raval, Will Riordan, Nabeel Nabulsi, Yiyun Huang, Brian Pittman, David Matuskey, Gustavo A. Angarita, Robin Bonomi, Sherry A. McKee, Ansel T. Hillmer, Kelly P. Cosgrove

**Affiliations:** 1 Department of Psychiatry,; 2 Yale Positron Emission Tomography (PET) Center, and; 3 Department of Radiology and Biomedical Imaging, Yale University, New Haven, Connecticut, USA.

**Keywords:** Clinical Research, Neuroscience, Addiction, Neuroimaging, Synapses

## Abstract

**BACKGROUND:**

Chronic alcohol use leads to synaptic dysfunction in preclinical studies. However, whether in vivo synaptic density deficits are found in people with alcohol use disorder (AUD) remains unclear.

**METHODS:**

Thirty-two people with AUD (*n* = 17 women; *n* = 15 men) and 29 control participants (*n* = 17 women; *n* = 12 men)completed 1 PET brain imaging scan with the radiotracer [^11^C]UCB-J, which binds to SV2A, a marker of synaptic density. The levels of synaptic density were quantified by estimating the nondisplaceable binding potential (*BP*_ND_) across 4 regions of interest: frontal cortex, striatum, hippocampus, and cerebellum.

**RESULTS:**

People with AUD were, on average (±SD), 43 ± 13 years of age, and most met the criteria for having mild or moderate AUD. The control participants were 37 ± 12 years of age. People with AUD had, on average, a 11% lower [^11^C]UCB-J *BP*_ND_ than did controls in the frontal cortex [*F*_(1,62)_ = 13.074, *P* < 0.001], striatum [*F*_(1,60)_ = 10.283, *P* = 0.002], and hippocampus [*F*_(1,60)_ = 5.964, *P* = 0.018], trending in the same direction in the cerebellum [*F*_(1,50)_ = 3.438, *P* = 0.070]. Among people with AUD, lower [^11^C]UCB-J *BP*_ND_ was significantly related to more drinks per drinking day, in the frontal cortex (*P* = 0.022) and striatum (*P* = 0.026). People with AUD performed worse on executive function than did controls (*P* = 0.020), but this was not related to [^11^C]UCB-J *BP*_ND_.

**CONCLUSION:**

Synaptic density deficits were evident, even in people with mild-to-moderate AUD, with larger deficits observed in those with greater drinking severity. These findings underscore the potential of synaptic restoration as a therapeutic target for AUD.

**FUNDING:**

NIH (U54AA027989, P01AA02747307, K01AA029706, and K24AA031345); UCB Pharma SA.

## Abstract 2

Synaptic density deficits are evident in people with alcohol use disorder and those with greater deficits show greater drinking severity. Synaptic density may serve as a molecular target for future therapeutic development.

## Introduction

Alcohol use is a severe public health problem and is responsible for a substantial part of the global burden of disease and mortality (1). A mounting body of preclinical literature has shown that alcohol has detrimental effects on synaptic transmission, synaptic function, synaptic plasticity, and overall behavior (2) by altering neurotransmitter release, receptor signaling, and gene expression in the brain (3, 4). These alcohol-induced neuroadaptations can result in neurocognitive dysfunction that can severely compromise an individual’s ability to maintain sobriety (5, 6). Although preclinical studies link synaptic dysfunction to poor brain health and neurodegeneration, the role of synaptic changes in clinical alcohol use disorder (AUD) remains poorly understood. This gap limits the development of novel treatments, despite the potential for synaptic restoration to serve as a therapeutic target in AUD.

A substantial body of preclinical studies have shown that chronic alcohol intake is associated with the loss of synapses in the cerebellum (7–9) and hippocampus (10), an early marker of neurodegeneration. Following a chronic binge drinking model in mice, another study showed aberrant synaptic pruning and substantial synaptic loss in the prefrontal cortex that was related to increased anxiety-like behavior (11). There is also evidence that lower synaptic density may contribute to neuronal dysfunction (12), particularly in the corticostriatal glutamate-GABA neurotransmitter imbalance and dopamine dysfunction (13), which are known to develop with chronic alcohol consumption and affect anxiety, mood, and withdrawal (14). Alcohol-induced impairments in synaptic plasticity that affect neurocognitive function in rats (15) persist even after 5 months of abstinence (16). However, another model suggests a potential recovery to control levels after prolonged abstinence (7). It is unknown if chronic alcohol use leads to lasting synaptic changes in humans. Findings in human postmortem studies are conflicting (17, 18), indicating a need to translate preclinical findings to living humans.

Lower gray-matter volume in corticolimbic brain regions, as measured by MRI, has been consistently reported in people with AUD relative to controls and is related to poorer neurocognition and affective (anxiety and depression) processing (19). Deficits in other markers of neuronal plasticity have also been observed in people with AUD and have been linked to poorer treatment outcomes (20–22). For example, lower brain volume and cortical thickness are related to a greater propensity to relapse rather than abstain from alcohol following treatment and to a greater magnitude of post-treatment alcohol consumption (20). Taken together, these findings suggest that decrements in neuromorphology may affect treatment outcomes for people with AUD. While these MRI studies provide valuable insight into the brain’s macrostructure, they do not directly assess synaptic density.

Advances in PET imaging have enabled in vivo quantification of synaptic density in the human brain using the radiotracer [^11^C]UCB-J, which binds to synaptic vesicle glycoprotein protein 2A (SV2A), a protein ubiquitously present in presynaptic vesicles (23) with a remarkably well-conserved stoichiometry to synapses (24). In nonhuman primates, in vivo SV2A radiotracer binding shows a strong positive correlation with ex vivo protein expression of SV2A and synaptophysin, a gold-standard marker of synaptic density (23, 25). Consequently, [^11^C]UCB-J binding serves as a reliable biomarker for synaptic density in the living human brain. We conducted a PET imaging study using [^11^C]UCB-J in individuals with and without AUD and collected clinical and cognitive assessments. Based on preclinical work, we hypothesized that people with AUD would have significantly lower synaptic density than controls and that this lower synaptic density would be associated with worse drinking outcomes and greater neurocognitive impairment.

## Results

### Study participant characteristics.

Individuals with AUD reported drinking on average 5 drinks/drinking day, 4 drinking days/week for 12 years (Table 1). At the time of the scan, all individuals with AUD met the National Institute on Alcohol Abuse and Alcoholism (NIAAA) heavy drinking criteria, except 5 participants, who volitionally reduced their drinking in the month prior to the scan (see Supplemental Methods for sensitivity analysis showing that removal of these participants did not change the results). Five people met the criteria for severe AUD based on the Diagnostic and Statistical Manual of Mental Disorders, 5th Edition (DSM-5), but most participants met the criteria for mild or moderate AUD. The controls were well matched with regard to sex, education level, and cigarette smoking status and reported on average less than 1 drink/drinking day and less than 1 drinking day/week for 10 years. The control participants tended to be younger than the individuals with AUD (mean age 37 vs. 43 years), however, age was not related to nondisplaceable binding potential (*BP*_ND_) values in any regions (*P* > 0.362). The participants self-identified as White (66%), Black (11%), Asian (8%), Hispanic or Latino (8%), American Indian/Alaska Native (2%), or race and/or ethnicity category not listed (5%). The injected dose, plasma-free fraction, mass, and specific activity of [^11^C]UCB-J did not differ significantly between groups (*P* > 0.05) (see Supplemental Methods for more details).

### Group differences in BP_ND_ values.

**Figure 1:**
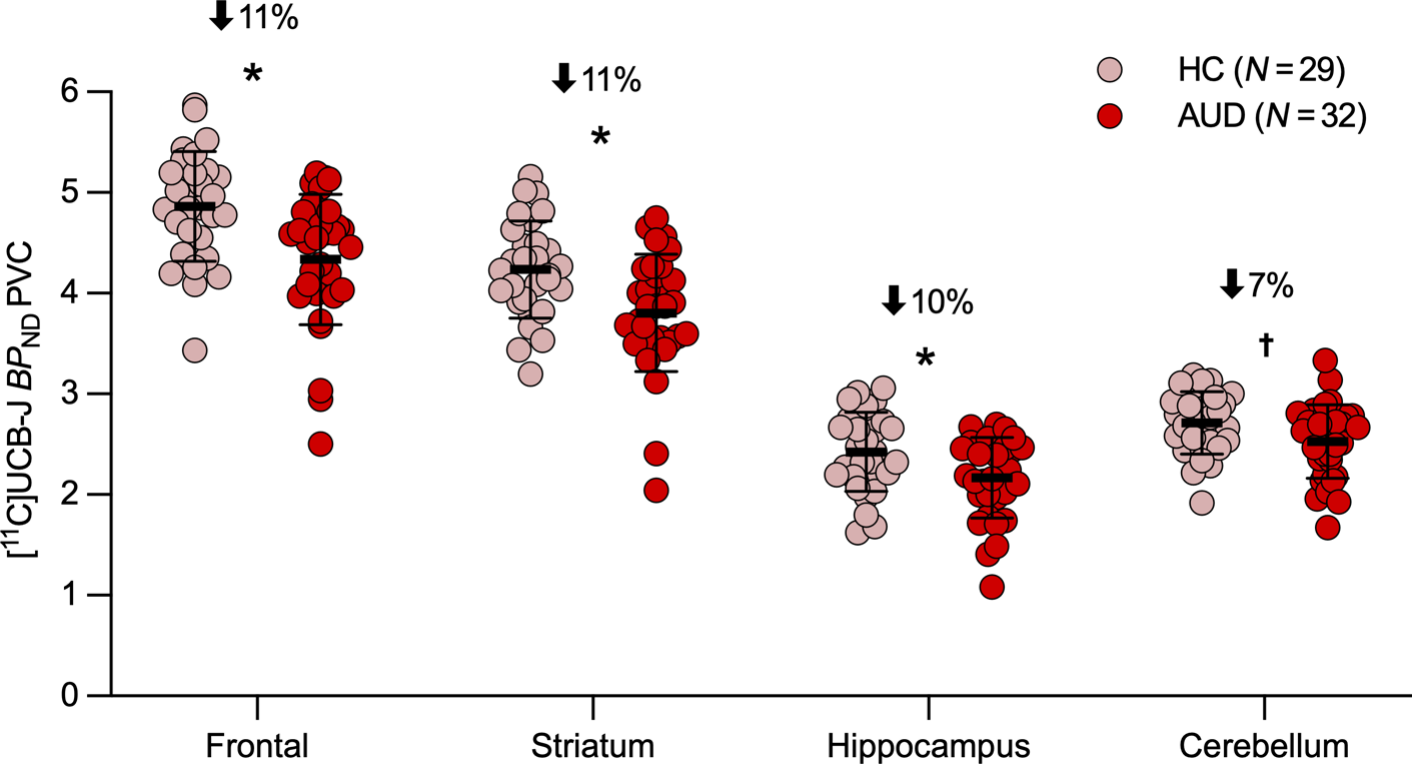
Synaptic density levels by diagnostic group. PVC *BP*_ND_ values were lower in 32 people with AUD (red dots) compared with values for 29 controls (healthy control [HC], light pink dots) in the frontal cortex, striatum, and hippocampus and trending in the same direction in the cerebellum, based on the mixed model. The percentage differences were 11%, 11%, 10%, and 7%, respectively. Data represent the mean ± SEM. *P < 0.05; ^†^*P* < 0.10.

**Figure 2:**
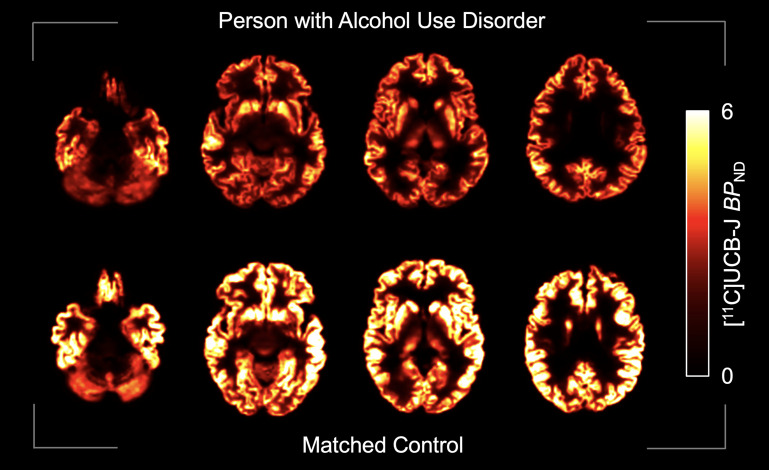
Synaptic density images by diagnostic group. Parametric images of PVC *BP*_ND_ maps from 2 representative participants who were similar to the group.

Consistent with our hypothesis, [^11^C]UCB-J *BP*_ND_ was significantly lower in people with versus those without AUD across all 4 brain regions [*F*_(1,57)_ = 11.193, *P* = 0.001; Figure 1]. This did not differ by sex [*F*_(1,57)_ = 0.012, *P* = 0.912; Supplemental Figure 2; supplemental material available online with this article; https://doi.org/10.1172/JCI199989DS1] Post hoc analysis revealed significant effects of AUD in the hippocampus [*F*_(1,60)_ = 5.964, *P* = 0.018], frontal cortex [*F*_(1,62)_=13.074, *P* < 0.001], and striatum [*F*_(1,60)_ = 10.283, *P* = 0.002] and trended in the same direction in the cerebellum [*F*_(1,50)_ = 3.438, *P* = 0.070]. *BP*_ND_ values were on average 11% lower in people with AUD than in controls across primary regions (Figure 1). Whole-brain *BP*_ND_ maps are shown (Figure 2), and *BP*_ND_ values for other brain regions are included in Supplemental Table 2, highlighting that the findings were region specific. Although the groups were matched on smoking status, inclusion of smoking status as a covariate in the model did not change the significance of the results.

### Clinical correlates of AUD.

**Figure 3:**
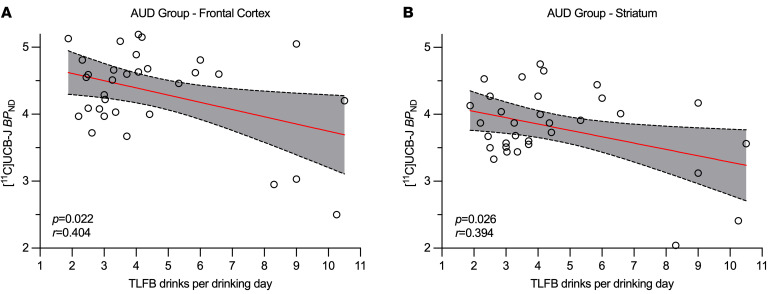
Synaptic density and drinking frequency. Among 32 people with AUD, lower *BP*_ND_ values (synaptic density) in the frontal cortex (**A**) (*P* = 0.022 uncorrected, *P* = 0.0514 FDR-corrected) and the striatum (**B**) (*P* = 0.026 uncorrected, *P* = 0.0514 FDR-corrected) were associated with more drinks per drinking day based on regression analyses. 95% confidence bands are shown.

In the AUD group, lower *BP*_ND_ values were related to more drinks per drinking day in the frontal cortex (*P* = 0.022, *r* = 0.404) and striatum (*P* = 0.026, *r* = 0.394; Figure 3). Trending relationships were observed between synaptic density and alcohol drinking quantity and alcohol use severity, such that lower *BP*_ND_ values were related to more grams of alcohol consumed per day (*P* = 0.064, *r* = 0.3330) and higher Alcohol Use Disorders Identification Test (AUDIT) (*P* = 0.069, *r* = 0.395) scores (Supplemental Figure 3).

Compared with controls, people with AUD performed worse on executive function (mean errors for AUD vs. control: 36 vs. 20, *P* = 0.020; Table 1) but not verbal learning and memory (*P* > 0.585). The number of errors on the executive function task was not related to *BP*_ND_ values in the 4 brain regions (*P* > 0.587). Performance on cognitive tasks was not related to Clinical Institute Withdrawal Assessment for Alcohol (CIWA-Ar) withdrawal scores (*P* > 0.26). Mood and anxiety scores assessed in this sample were not related to *BP*_ND_ values across all brain regions for all participants or within the AUD group only.

## Discussion

In this study, we investigated synaptic density in people with AUD in vivo with [^11^C]UCB-J PET imaging. We found an approximately 11% lower synaptic density in the frontal cortex, striatum, and hippocampus of people with AUD compared with controls. Among people with AUD, lower synaptic density in the frontal cortex and striatum was related to a higher number of drinks per drinking day and trended in the same direction for alcohol drinking quantity and alcohol use severity. Taken together, these findings suggest that chronic alcohol use contributes to dose-dependent synaptic loss and that synaptic restoration may be a promising treatment strategy for AUD.

This study translated preclinical findings to living humans with AUD, filling a crucial gap in the literature. Preclinical studies have shown that synaptic density in the hippocampus (10) and cerebellum (7–9) was lower in rodents chronically exposed to alcohol versus controls, with recovery of synaptic density in a subset of chronically alcohol-treated rodents compared with controls (7). Another study using human postmortem tissue showed evidence of frontal cortex synaptic loss in people with AUD relative to controls (17). Our finding of lower synaptic density in people with AUD relative to controls is consistent with prior work (26), adds to the limited knowledge on the neurobiological mechanisms underlying synaptic impairment in people with AUD, and suggests that targeting the restoration of synaptic density could improve AUD-related impairments in brain function.

Among people with AUD, lower synaptic density was associated with higher drinking frequency (more drinks per drinking day), specifically in the frontal cortex and striatum. We found a similar relationship between drinking quantity and AUDIT scores in the striatum, although these results were at the trend level. These findings suggest that the synaptic deficit in people with AUD may be dose dependent, such that individuals who drink more could be “losing” more presynaptic vesicles or synapses in these brain regions in a coordinated fashion. The frontal cortex and striatum are part of the corticolimbic circuit, and neuroimaging studies have largely converged on the dysregulation of neurotransmission in corticolimbic brain regions in people with AUD and other substance use disorders (27). Alterations in neurotransmission (such as dampening of synaptic excitation and reduction of synaptic plasticity) constitute one way in which chronic alcohol use can disrupt synaptic plasticity, although a direct mechanism has not yet been established (28). Other prevailing potential mechanisms include synapse loss through microglia-mediated synaptic pruning (29) and disruption of synaptic protein structure and function (30). Our findings suggest that individuals who drink more alcohol have lower synaptic density in the primary brain regions selected, which may affect higher-order brain functions due to loss of synapses in these brain regions.

It is important to note that most (27 of 32) of the participants with AUD in this study had mild-to-moderate AUD. This limited variability in our alcohol-related measures such as drinking frequency. Most people drank between 2 and 7 drinks per day, and most AUDIT scores were between 3 and 12, consistent with low-to-moderate risk of hazardous drinking on average. This inherently limited the dynamic range for evaluating the effect of AUD severity on synaptic density. However, our findings suggest that, even in a population representative of individuals with mild-to-moderate AUD, alcohol-induced neuroadaptations (synaptic loss) are observed.

This study has several strengths, including using well-validated SV2A imaging methods to investigate synaptic density in people with AUD in vivo. Similar results were observed in a subset of participants (*n* = 38) with regional volume of distribution (*V*_T_) (see Supplemental Methods), supporting the use of noninvasive estimation of [^11^C]UCB-J *BP*_ND_ to provide a larger and more robust sample. [^11^C]UCB-J *V*_T_ in the centrum semiovale was 8% lower in people with AUD compared with controls (see Supplemental Methods). While not statistically significant, this trend may reflect true biologically lower SV2A levels in this pseudo-reference region, however, such an effect would cause an overestimation of [^11^C]UCB-J *BP*_ND_ in the AUD group and consequently underestimate the group differences in [^11^C]UCB-J *BP*_ND_, further supporting the strength of our primary findings with this outcome measure. Partial volume correction for gray-matter atrophy was another strength because we observed lower gray-matter volume in people with AUD versus those without AUD (see Supplemental Methods), consistent with large samples of people who drink at mild-to-moderate levels (31).

This study also has limitations that can be addressed with future studies. We expected to find associations between synaptic density and cognitive performance in people with AUD. While we observed lower synaptic density and poorer cognitive performance on an executive function task in people with AUD, we did not observe a direct relationship between synaptic density and cognitive function, suggesting that other synaptic proteins or processes may be more directly linked with neurocognition. [^11^C]UCB-J targets SV2A, which is ubiquitously expressed in presynaptic vesicles across neuronal populations, failing to capture the postsynaptic proteins and neuron population–specific effects observed in animal research (7). Prior reports of a relationship between SV2A expression and neurocognition in healthy individuals and those with psychiatric conditions have been mixed (32–34). Given the extensive literature showing greater vulnerability in brain deficits in women with AUD (35–38), we also examined preliminary sex differences. Our findings indicate that men and women with AUD had similar synaptic deficits relative to their control counterparts in our mild-to-moderate AUD sample. Future studies should extend this work to determine if there is an interaction between sex, AUD severity, and synaptic density in populations with more severe AUD. Another limitation is that it is not possible to determine whether the observed synaptic deficits were a consequence or a precursor of AUD, given the observational, case-control design of the study. Future studies should also determine, using PET imaging, the extent, if any, of synaptic density recovery with alcohol abstinence.

Taken together, our findings show that AUD contributes to synaptic neurodegeneration and that lower synaptic density is related to greater drinking severity. We demonstrate that dysfunction in corticolimbic brain regions in people with AUD could have a molecular basis in synapse density loss, such that more drinking is related to greater synapse loss. This work has important implications for the future therapeutic development of more effective treatment targets for AUD that are aimed at synaptic function, such as with psychedelic compounds, which have shown therapeutic promise (39). Restoration of the loss of synapses could in turn restore vulnerable brain functions and alleviate AUD symptoms.

## Methods

### Sex as a biological variable

Men and women were included in all parts of the study.

### Participants

A total of 61 participants (*n* = 32 people with AUD: 17 women, 15 men; and *n* = 29 controls: 17 women, 12 men) were recruited from the local population (Table 1). All participants completed one [^11^C]UCB-J PET scan and one MRI scan.

Individuals with AUD met either DSM-IV or DSM-5 criteria for alcohol dependence or AUD, respectively, with no other substance use disorder (except tobacco/nicotine) and no current or past marked medical or neurological disorders. To minimize the risk of withdrawal complications (40, 41), participants were excluded if they reported undergoing repeated (defined as 4 or more in the past 5 years) medicated alcohol detoxifications (e.g., requiring benzodiazepines), or showed substantial withdrawal symptoms at intake, as assessed by the CIWA-Ar (42). Participants with AUD were had abstained overnight from alcohol, as confirmed by breathalyzer on the morning of the PET scan.

The controls had no history of major medical disorders or head trauma and did not meet the DSM-5 criteria for current or past substance use disorder (excluding tobacco/nicotine). The control participants were required to report drinking fewer than 5 alcohol-containing drinks per week with no heavy drinking days in the last 30 days, based on the NIAAA heavy drinking criteria (5 drinks/day or 15 drinks/week for men and 4 drinks/day or 8 drinks/week for women).

For all female participants, negative pregnancy tests were required during screening and prior to radiotracer administration on the day of the scan. All participants were cisgender (the gender identity matched the biological sex).

### Clinical, cognitive, and biological assessments

During screening and on the PET scan day, alcohol use severity was assessed with the AUDIT (43). Alcohol use over the previous month was documented using the timeline follow-back (TLFB) assessment method (44).

On the day of the PET scan, participants completed mood (Center for Epidemiologic Studies Depression Scale [CES-D]) (45) and anxiety (Spielberger’s State-Trait Anxiety Index [STAI]) (46) questionnaires, as well as a computerized cognitive battery (Cogstate) (47) assessing executive function and verbal learning and memory.

For the executive function (set-shifting) task, participants learned to correctly identify if a playing card met an underlying rule set related to the card color (black or red) or number using trial-and-error strategies. Visual and auditory feedback was given, and the rule sets changed over time. The total number of errors made was calculated. For the verbal learning and memory (international shopping list) task, participants were asked to remember a list of items on 3 consecutive trials, and the total number of items recalled immediately (learning) and after a 30-minute delay (memory) was calculated.

### Imaging data acquisition, processing, and analysis

[^11^C]UCB-J was synthesized as reported previously (48). Participants received a 1-minute intravenous bolus injection of 541 ± 183 MBq [^11^C]UCB-J via an automated infusion pump (Harvard PHD 22/2000, Harvard Apparatus) and were scanned for 90 minutes on a high-resolution research tomograph (HRRT) (Siemens, Medical Solutions). Prior to radiotracer administration, a 6-minute ^137^Cs transmission scan was acquired for attenuation correction of the emission data. For a subset of participants (*n* = 38, 22 with AUD; Supplemental Tables 1 and 3), an arterial catheter was placed contralaterally to the [^11^C]UCB-J injection site in the radial artery to determine whether there was a need for arterial sampling for this population. The metabolite-corrected arterial input function was measured as previously described (23). Our analysis confirmed the validity of the centrum semiovale as a reference region and led to the use of *BP*_ND_ as the primary outcome measure (see Supplemental Methods).

A T1-weighted structural MRI was acquired for coregistration of PET images and definition of anatomical regions of interest (ROIs), with a 3T Trio/Prisma Scanner (Siemens Medical Systems) using a magnetization-prepared rapid gradient-echo sequence (TE = 2.81 ms; TI = 1,100 ms, TR = 2,530 ms, FA = 7°, 1 mm^3^ isotropic resolution). Structural MR images were processed using a computational anatomy toolbox (CAT12) (49) and FreeSurfer, version 6.0 (http://surfer.nmr.mgh.harvard.edu/). Brain extraction was performed with CAT12, and the resulting images were input into the FreeSurfer pipeline for intensity normalization, Talairach transformation, and tissue segmentation. Cortical and subcortical parcellation was conducted using the Desikan-Killiany atlas to generate subject-specific ROIs.

Dynamic list-mode PET data were binned into frames of increasing length up to 5 minutes and reconstructed with MOLAR (50), correcting for head motion (Polaris Vicra Optical Tracking System, NDI Systems), attenuation, scatter, randoms, and dead time. The first 10 minutes of PET data were registered to the subject-specific, T1-weighted MRI using a mutual information algorithm with 6 degrees of freedom (FLIRT, FSL 3.2; Analysis Group; FMRIB). Time-activity curves of radioactivity concentration were extracted from the ROIs defined using FreeSurfer-derived parcellations and masked with subject-specific gray-matter segmentation. A priori ROIs included the frontal cortex, striatum, hippocampus, and cerebellum, based on our previous findings (35, 51) and the critical role of these regions in alcohol-induced neurodegeneration (52, 53).

The primary PET outcome measure was the *BP*_ND_, which serves as a measure of synaptic density in vivo. Regional *BP*_ND_ values were calculated using the simplified reference tissue model 2 with a group-specific fixed *k*_2_′ to account for inherent group differences and the centrum semiovale as a reference region, as previously described (23) (see Supplemental Methods for more details). Iterative Yang partial volume correction (PVC) (54, 55) was applied to account for potential effects of gray-matter atrophy on PET outcomes, based on group-level differences in gray-matter volume (Supplemental Figure 1 and Supplemental Table 4).

#### Sex as a biological variable.

Given the known sex differences showing that women with AUD have a greater vulnerability to brain deficits (35–38), we also ran a linear mixed model (identical to the one described below), adding sex as a between-subjects factor to test for preliminary sex-specific contrasts.

### Statistics

A *P* value of less than 0.05 was considered significant. Data for Figure 1 is presented as Mean ± SEM. Figures 2 and 3 show individual data values.

#### Linear mixed model.

Regional [^11^C]UCB-J *BP*_ND_ values were statistically analyzed with a linear mixed model to test the null hypothesis of no difference in *BP*_ND_ values between diagnostic groups. The model included the diagnostic group (AUD vs. controls) as a between-subjects factor and ROI (frontal cortex, striatum, hippocampus, and cerebellum) as a within-subject repeated factor. All interactions were modeled. Post hoc linear contrasts were generated to examine regional differences in *BP*_ND_ values between individuals with AUD and controls, based on a priori hypotheses.

#### T tests.

Group differences in cognitive performance, mood, and anxiety were assessed using independent-sample 2-tailed *t* tests comparing AUD with controls.

#### Linear regressions.

Linear regressions examined relationships between regional *BP*_ND_ values and primary clinical correlates of interest (based on refs. 35, 51): TLFB (drinks per drinking day), AUD severity (AUDIT), and neurocognition (executive function, verbal learning and memory) across regions. Exploratory linear regressions were conducted between regional *BP*_ND_ values and secondary clinical correlates of interest: mood (CES-D) and anxiety (STAI). Neurocognition, mood, and anxiety measures were selected on the basis of a priori hypotheses and our prior reports showing relationships with PET outcome measures (56–58). Regression analyses were conducted separately for the full sample and the AUD group. Secondary regressions were not corrected for multiple comparisons because of their exploratory nature. All statistical analyses were performed using SPSS, version 29.

### Study approval

Written informed consent was obtained from all participants prior to enrollment in this study. All study procedures were approved by the Yale Human Investigation Committee and the Yale-New Haven Hospital Radiation Safety Committee and complied with guidelines for the Protection of Human Subjects of Research and Ethical Principles.

### Data availability

The deidentified PET and behavioral data for this study are available through the NIAAA Data Archive repository (doi: 10.15154/7pt9-ey02). Values for all data points shown in graphs and reported means in tables can be found in the Supporting Data Values file.

## Author contributions

This project was conceptualized by KPC and SAM. Data collection, data extraction, and project administration were performed by YZ, ATH, WR, and KPC. Formal analysis was performed by YZ, NRR, and ATH. The first draft of the manuscript was written by YZ and NRR and all other authors (WR, NN, YH, BP, DM, GAA, RB, SAM, ATH, and KPC) reviewed drafts of the manuscript and approved the final manuscript.

## Funding support

This work is the result of NIH funding, in whole or in part, and is subject to the NIH Public Access Policy. Through acceptance of this federal funding, the NIH has been given a right to make the work publicly available in PubMed Central.

- NIH grants U54AA027989 and P01AA02747307 (to SAM), K01AA029706 (to YZ), and K24AA031345 (to KPC).
- UCB Pharma SA (to YH).

## Supplementary Material

Supplemental data

ICMJE disclosure forms

Supporting data values

## Figures and Tables

**Table 1:**
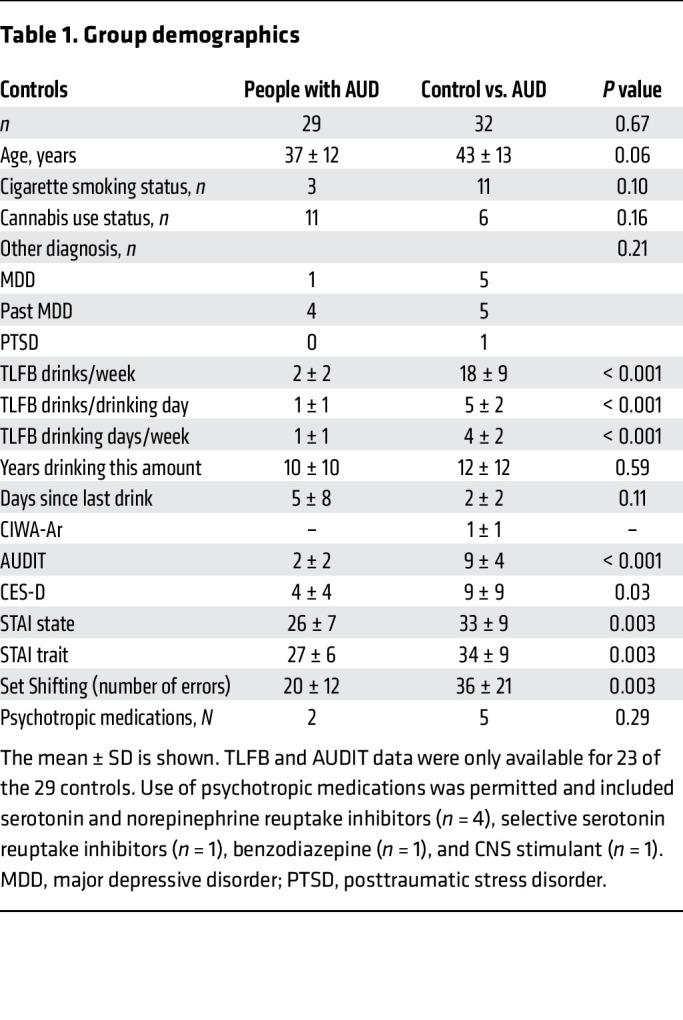
Group demographics

## Version 1 01/13/2026

In-Press Preview

## Version 2 04/01/2026

Electronic publication
