# Supplementary material for: Higher drinking frequency corresponds to lower synaptic density in people with alcohol use disorder
Source: J Clin Invest. 2026 Jan 13;136(7):e199989. doi: 10.1172/JCI199989 (PMC13038192; doi:10.1172/JCI199989)
Supplement: Supplemental data [file jci-136-199989-s051.pdf]

SUPPLEMENTARY INFORMATION

People with alcohol use disorder have lower synaptic density that is associated with greater drinking severity

Zakiniaeiz, et al.

TABLE OF CONTENTS:

Methods: Arterial Input Modeling and Reference Region Validation (Centrum Semiovale)

Methods: Estimation of Grey-Matter Volume (GMV)

Methods and Results: Additional Analyses to Examine Potential Confounds

- Age
- Days since last drink
- Age at first drink
- Subjects who volitionally reduced drinking
- AUD severity per DSM-5 criteria
- World Health Organization (WHO) risk drinking levels
- Cannabis use
- Benzodiazepine use
- Depression

Table S1: Subject Demographic Table for Subsample with V_T Data

Table S2: Mean PVC BP_{ND} in Primary and Secondary ROIs

Table S3: Mean PVC V_T in Primary and Secondary ROIs

Table S4: Mean non-PVC BP_{ND} and V_T in Primary ROIs

Figure S1: Grey-Matter Volume Differences

Figure S2: Mean BP_{ND} by Diagnosis and Sex

Figure S3: Relationship between UCB-J BP_{ND} and drinking amount and AUD severity

Methods: Arterial Input Modeling and Reference Region Validation (Centrum Semiovale)

For the subset of participants with arterial blood data (n = 38, **Table S1**), kinetic modeling was performed using a one-tissue compartment model (1TCM) to estimate the total distribution volume (V_T) across regions of interest. The metabolite-corrected arterial input function was used for this analysis.

To validate the use of the centrum semiovale as a reference region for BP_{ND} quantification, we examined whether there were any group differences in V_T and the clearance rate (k_2) values within this region. Group comparisons were conducted using unpaired two-tailed parametric *t*-tests. No significant group difference was observed in the centrum semiovale V_T values between the AUD (3.83 ± 0.13 mL/cm^3) and control groups (4.14 ± 0.17 mL/cm^3) (t=1.49, p=0.146), supporting its use as a reference region for BP_{ND} estimation. However, the clearance rate from the reference region (k_2') differed between groups. The mean k_2 for the centrum semiovale was 0.027 ± 0.001 min^{-1} in controls and 0.029 ± 0.001 min^{-1} in the AUD group (t=2.01, p=0.051). Based on this result, group-specific fixed k_2' values (controls = 0.027 min^{-1}; AUD = 0.029 min^{-1}) were used in the SRTM2 model for the primary BP_{ND} analysis.

We observed a significant main effect of diagnostic group on regional V_T values, consistent with the primary BP_{ND} findings in our *a priori* regions including the frontal cortex, striatum, hippocampus, and cerebellum. Specifically, a similar group difference was detected in this subset, with lower V_T observed in the AUD group compared to controls (F(1,34)=10.614, p=0.003).

Methods: Estimation of Grey-Matter Volume (GMV)

Structural T1-weighted MR images were first processed using the Computational Anatomy Toolbox (CAT12), which performed brain extraction and produced skull-stripped images. These images were then input into FreeSurfer version 6.0 (http://surfer.nmr.mgh.harvard.edu/), which completed the full recon-all pipeline including intensity normalization, Talairach transformation, cortical surface reconstruction, and subcortical segmentation.

GMV was estimated using regional volumes extracted from FreeSurfer's *aseg.stats* output file. For each subject, the total grey-matter volume was computed by summing the volumes of relevant cortical and subcortical grey-matter structures. To account for inter-individual differences in head size, all regional GMV values were normalized to the estimated total intracranial volume (eTIV), also provided in the *aseg.stats* file. These normalized values were used in group-level comparisons.

Methods and Results: Additional Analyses to Examine Potential Confounds

Age

The age by diagnosis interaction in the mixed model was not significant ($F_{(2,59)}$=1.477, p=0.237) for BP_{ND}, suggesting that age did not influence the results.

Days since last drink

Data were available for 27 out of 32 participants with AUD. Days since last drink was not related to BP_{ND} in any of the four primary ROIs, 0.483<p's<0.976.

Age at first drink

Data were available for 22 out of 32 participants with AUD. Age at first drink was not related to BP_{ND} in any of the four primary ROIs, 0.445<p's<0.988.

Withdrawal

CIWA score was not related to BP_{ND} in any of the four primary ROIs, 0.137<p's<0.620

Subjects who volitionally reduced drinking

Because five participants with AUD volitionally reduced their drinking prior to scanning, a sensitivity analysis was conducted to determine if these participants influenced the results. Removing these five (only three for V_T analysis) participants did not change the results of the mixed model ($F_{(1,52)}$=11.284, p=0.001) for BP_{ND} and $F_{(1,31)}$=11.245, p=0.002) for V_T.

World Health Organization (WHO) risk drinking levels

We were able to obtain WHO risk drinking categorical levels on all 31 participants with AUD. WHO risk drinking levels were related to BP_{ND} ($F_{(3,28)}$=2.299, p=0.099) at the trend level across all 4 ROIs. *Post-hoc* analyses by region revealed that people with AUD who drink at moderate

risk levels have significantly lower BP_{ND} than those who drink at low risk levels in the striatum (p=0.016) and frontal cortex (p=0.032).

AUD severity per DSM-5 criteria

All individuals with AUD met either DSM-IV or DSM-5 criteria for alcohol dependence or AUD, respectively. Individuals who met DSM-IV criteria were converted to DSM-5 to obtain DSM-5 equivalent severity measures when possible (when we have enough information in the itemized symptoms to appropriately estimate severity). We were able to obtain severity for 26 of 32 participants with AUD (12 mild, 9 moderate, 5 severe). AUD severity in these 26 participants was not related to BP_{ND} ($F_{(2,23)}$=1.396, p=0.268).

Cannabis use

Including cannabis use status in the mixed model did not change the results ($F_{(1,57)}$=10.102, p=0.002) for BP_{ND} and $F_{(1,35)}$=9.568, p=0.004) for V_T.

Benzodiazepine use

Because one participant with AUD was on a stable dose of a benzodiazepine prior to scanning, a sensitivity analysis was conducted to determine if this participant influenced the results. Removing this participants did not change the results of the mixed model ($F_{(1,56)}$=11.314, p=0.001) for BP_{ND}. This subject was not included in the V_T analyses.

Depression

Removing the participants with current MDD (five in the AUD group and one in the control group for BP_{ND} analysis and three in the AUD group for V_T analysis) did not change the results of the mixed model ($F_{(1,54)}$=10.418, p=0.002) for BP_{ND} and $F_{(1,31)}$=9.247, p=0.005) for V_T. Further,

depression scores on the Center for Epidemiologic Studies Depression Scale (CES-D; $0.125 < p$'s < 0.951) and Hamilton Depression Scale ($0.151 < p$'s < 0.946) were not related to BP_{ND}.

	Controls	People with AUD	Control vs. AUD *p*-value
**N**	16	22	0.40
**Age, years**	39 ± 12	42 ± 14	0.53
**Cigarette Smoking Status, N**	3	9	0.53
**Cannabis Use Status, N**	7	4	0.09
**Other Diagnosis, N**			0.06
MDD	0	3	
Past MDD	0	4	
PTSD	0	1	
**TLFB Drinks/week**	1 ± 2	17 ± 8	**<0.001**
**TLFB Drinks/drinking day**	1 ± 1	4 ± 2	**<0.001**
**TLFB Drinking days/week**	1 ± 1	4 ± 2	**<0.001**
**Years Drinking this amount**	16 ± 13	14 ± 13	0.79
**Days Since Last Drink**	8 ± 11	2 ± 2	0.06
**CIWA-Ar**	—	1 ± 1	—
**AUDIT**	2 ± 2	9 ± 5	**<0.001**
**CES-D**	5 ± 5	11 ± 11	0.19
**STAI State**	27 ± 8	35 ± 10	**0.03**
**STAI Trait**	27 ± 6	34 ± 9	**0.01**
**Set Shifting (number of errors)**	22 ± 17	36 ± 18	0.08
**Psychotropic Medications, N**	0	3	0.12

Table S1: Subject Demographic Table for Subsample with V_T Data. Means ± SD shown. TLFB and AUDIT data only available for 9 of 16 controls. Cognitive data only available for 8 out of 16 controls and 18 out 22 people with AUD. *Abbreviations*: AUDIT: Alcohol Use Disorders Identification Test; *CES-D*: Center for Epidemiologic Studies Depression Scale; *CIWA-A*r: Clinical Institute Withdrawal Assessment for Alcohol Revised; *MDD*: Major Depressive Disorder; *N/A*: Not Available due to missing data; *PTSD*: Post-traumatic Stress Disorder; *SD*: standard deviation, *STAI:* Spielberger's State-Trait Anxiety Index, *TLFB*: Timeline Followback.

		HC	AUD	Percent Difference	Effect Size (Cohen's *d*)
**Primary ROIs**	**Hippocampus**	2.42 ± 0.07	2.17 ± 0.07	10%	0.55
	**Frontal Cortex**	4.86 ± 0.10	4.34 ± 0.11	11%	0.88
	**Striatum**	4.24 ± 0.09	3.80 ± 0.10	11%	0.64
	**Cerebellum**	2.71 ± 0.06	2.53 ± 0.06	7%	0.80
**Secondary ROIs**	**Amygdala**	3.73 ± 0.11	3.70 ± 0.09	1%	0.06
	**ACC**	5.06 ± 0.12	4.79 ± 0.16	5%	0.34
	**Caudate**	3.57 ± 0.09	3.31 ± 0.10	7%	0.47
	**Insula**	4.49 ± 0.11	4.29 ± 0.10	4%	0.34
	**Occipital Cortex**	4.59 ± 0.10	4.20 ± 0.13	9%	0.61
	**OFC**	4.82 ± 0.11	4.59 ± 0.13	5%	0.33
	**Parietal Cortex**	5.08 ± 0.12	4.69 ± 0.18	8%	0.45
	**PCC**	5.11 ± 0.11	4.78 ± 0.16	6%	0.44
	**PFC**	4.95 ± 0.11	4.57 ± 0.11	8%	0.62
	**Putamen**	4.68 ± 0.10	4.32 ± 0.17	8%	0.46
	**Temporal Cortex**	4.84 ± 0.11	4.61 ± 0.13	5%	0.34
	**Thalamus**	2.43 ± 0.06	2.40 ± 0.07	1%	0.08
				***p*-value**	
**PET Injection Parameters**	**Plasma Free Fraction (fp)**	0.29 ± 0.008	0.27 ± 0.005	0.07	
	**Injected Dose (MBq)**	550 ± 32	532 ± 34	0.70	
	**Specific Activity (MBq/mol)**	211 ± 20	209 ± 20	0.94	

Table S2: Mean PVC BP_{ND} Values by Diagnosis for primary and secondary ROIs and PET Scan Injection Parameters. Means ± SEM shown. Primary regions identified *a priori* for statistical analysis and partial-volume corrected (PVC). Percent differences between controls and people with AUD are shown. Effect sizes are shown based on uncorrected, independent samples *t*-tests between groups. *Abbreviations*: Anterior Cingulate Cortex (ACC), Orbitofrontal Cortex (OFC), Posterior Cingulate Cortex (PCC), Prefrontal Cortex (PFC), SEM = standard error of the mean.

		HC	AUD	Percent Difference	Effect Size (Cohen's *d*)
**Primary ROIs**	**Hippocampus**	14.6 ± 0.6	12.8 ± 0.5	12%	0.77
	**Frontal Cortex**	24.3 ± 0.8	21.5 ± 1.0	11%	0.69
	**Striatum**	21.8 ± 0.7	19.0 ± 0.9	13%	0.74
	**Cerebellum**	15.5 ± 0.5	13.8 ± 0.4	11%	0.83
**Secondary ROIs**	**Amygdala**	20.0 ± 0.9	17.9 ± 0.7	11%	0.61
	**ACC**	25.3 ± 0.9	22.4 ± 0.7	10%	0.84
	**Caudate**	19.1 ± 0.7	16.6 ± 0.8	10%	0.72
	**Insula**	23.0 ± 0.8	20.2 ± 0.8	10%	0.78
	**Occipital Cortex**	23.4 ± 0.8	20.6 ± 1.0	10%	0.69
	**OFC**	24.2 ± 0.9	21.3 ± 0.8	10%	0.79
	**Parietal Cortex**	25.5 ± 0.8	22.3 ± 1.2	10%	0.69
	**PCC**	25.5 ± 0.9	22.6 ± 0.8	10%	0.79
	**PFC**	24.8 ± 0.8	21.9 ± 1.0	10%	0.71
	**Putamen**	23.8 ± 0.8	20.7 ± 1.0	10%	0.72
	**Temporal Cortex**	24.5 ± 0.8	21.7 ± 0.8	10%	0.78
	**Thalamus**	14.1 ± 0.5	12.9 ± 0.5	10%	0.58
				***p*-value**	
**PET Injection Parameters**	**Plasma Free Fraction (fp)**	0.29 ± 0.014	0.26 ± 0.007	0.08	
	**Injected Dose (MBq)**	549 ± 47	520 ± 43	0.65	
	**Specific Activity (MBq/mol)**	213 ± 34	199 ± 18	0.70	

Table S3: Mean PVC V_T Values by Diagnosis for primary and secondary ROIs and PET Scan Injection Parameters. Means ± SEM shown. Primary regions identified *a priori* for statistical analysis and partial-volume corrected (PVC). Percent differences between controls and people with AUD are shown. Effect sizes are shown based on uncorrected, independent samples *t*-tests between groups. *Abbreviations*: Anterior Cingulate Cortex (ACC), Orbitofrontal Cortex (OFC), Posterior Cingulate Cortex (PCC), Prefrontal Cortex (PFC), SEM = standard error of the mean.

		HC	AUD	Percent Difference	Effect Size (Cohen's *d*
BP_{ND}	**Hippocampus**	2.25 ± 0.06	2.06 ± 0.07	8%	0.51
	**Frontal Cortex**	3.96 ± 0.08	3.54 ± 0.09	11%	0.88
	**Striatum**	3.79 ± 0.08	3.47 ± 0.09	8%	0.70
	**Cerebellum**	2.56 ± 0.05	2.41 ± 0.06	6%	0.49
V_T	**Hippocampus**	13.8 ± 0.5	12.1 ± 0.5	12%	0.77
	**Frontal Cortex**	20.4 ± 0.7	18.1 ± 0.8	11%	0.69
	**Striatum**	19.9 ± 0.7	17.3 ± 0.8	13%	0.76
	**Cerebellum**	14.9 ± 0.5	13.2 ± 0.4	11%	0.84

Table S4: Mean non-PVC BP_{ND} and V_T Values by Diagnosis for primary ROIs. Means ± SEM shown. Primary regions identified *a priori* for statistical analysis and non-partial-volume corrected (non-PVC). Percent differences between controls and people with AUD are shown. Effect sizes are shown based on uncorrected, independent samples *t*-tests between groups. *Abbreviations*: SEM = standard error of the mean.

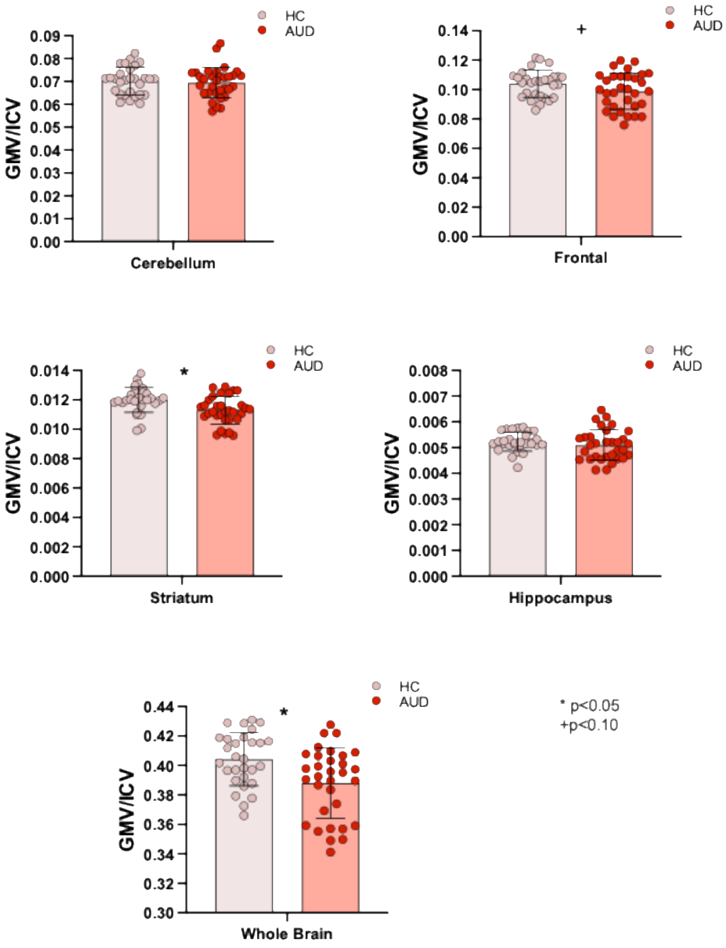

Figure S1: Grey-Matter Volume (GMV) Differences. GMV is normalized for total intracranial volume (ICV) per brain region and in the whole brain. Mean and SEM shown. HC=Healthy Control.

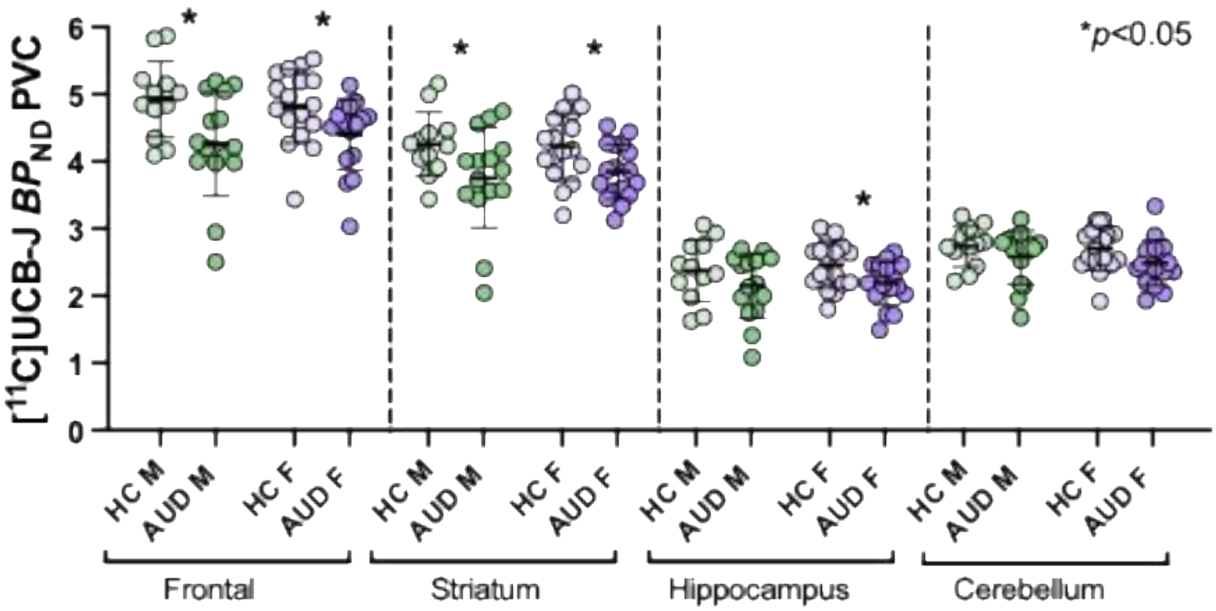

Figure S2: Mean BP_{ND} by Diagnosis and Sex. Levels of a SV2A BP_{ND} are lower in women with AUD (dark purple dots) compared to control men (light purple dots) in frontal cortex, striatum, and hippocampus. Levels of a SV2A BP_{ND} are lower in men with AUD (dark green dots) compared to control (HC) men (light green dots) in frontal cortex and striatum. Means and SEM shown. *indicates $p<0.05$.

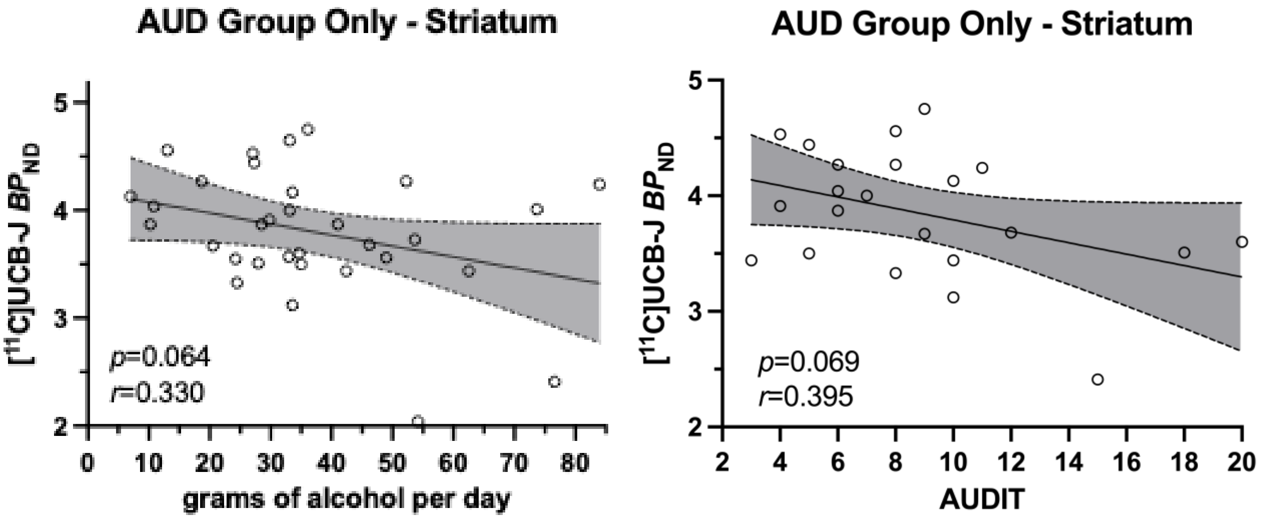

Figure S3: Relationship between UCB-J BP_{ND} and grams of alcohol perday and AUD severity. Lower SV2A BP_{ND} (synaptic density) in the striatum is associated with more drinking (grams of alcohol per day) and greater alcohol severity (higher AUDIT score) and at a trend level (p=0.064 uncorrected, p=0.234 FDR-corrected; p=0.069 uncorrected, p=0.275 FDR-corrected, respectively). 95% confidence bands shown.
